# Biofunctionalization of Magneto-Plasmonic Fe_3_O_4_@SiO_2_-NH_2_-Au Heterostructures with the Cellulase from *Trichoderma reesei*

**DOI:** 10.3390/molecules30030756

**Published:** 2025-02-06

**Authors:** Anna Tomaszewska, Magdalena Kulpa-Greszta, Oliwia Hryców, Klaudia Niemczyk, Renata Wojnarowska-Nowak, Daniel Broda, Robert Pazik

**Affiliations:** 1 Faculty of Biotechnology, Collegium Medicum, University of Rzeszow, Pigonia 1, 35-310 Rzeszow, Poland; atomaszewska@ur.edu.pl (A.T.); mkulpa@ur.edu.pl (M.K.-G.); oliwia.hrycow@op.pl (O.H.); klaudia.niemczyk1@gmail.com (K.N.); 2 Institute of Materials Engineering, College of Natural Sciences, University of Rzeszow, Pigonia 1, 35-310 Rzeszow, Poland; rwojnarowska@ur.edu.pl

**Keywords:** cellulase, *Trichoderma reesei*, immobilization, ferrite heterostructures, magneto-plasmonic nanomaterials

## Abstract

The study focuses on the synthesis of Fe_3_O_4_@SiO_2_-NH_2_-Au heterostructures with magneto-plasmonic properties composed of well-defined cubic Fe_3_O_4_ cores (79 nm) covered with 10 nm silica shell and gold nanoparticles (8 nm) fabricated on silica shell. The surface-anchored MHDA (16-mercaptohexadecanoic acid) linker facilitated cellulase bioconjugation, which was confirmed through Raman spectroscopy. The presence of gold nanoparticle islands on the heterostructure enabled surface-enhanced Raman scattering (SERS), demonstrating the potential for bioactive substance identification. Immobilization of cellulase allowed for pH enhancement and enzyme thermal stability. The optimal pH shifted from 4.0 (free enzyme) to 6.0 while thermal stability increased by 20 °C. The immobilized cellulase kept its 49% activity after five hydrolysis cycles, compared to significantly lower activity for free cellulase. The proposed heterostructures for cellulase immobilization demonstrate potential for practical applications.

## 1. Introduction

Cellulases are a group of highly important enzymes playing a crucial role in the bioconversion of cellulose-containing biomass into fermentable sugars [1,2]. These enzymes are produced by a variety of microorganisms such as fungi [3,4], bacteria [5], microalgae [6] as well as archaea [7]. Their vast range of applications covers biofuels, food, cosmetics, detergents, pulp, paper industry [8], and others. For instance, in the biofuel process production cellulase enzymes are of key meaning for the hydrolysis of lignocellulosic biomass and release of monomeric sugars [9], while in the textile industry they can be used for fiber modification by removing fuzz fibers and pills or textile softening [10]. In contrast, in paper production, cellulase is expected to enhance fiber–fiber bond strength [11]. Cellulase, especially of bacterial origin, is considered ecofriendly biomaterial with potential for biodegradation [12]. Therefore, the use of such enzymes in cosmetic applications has emerged in recent years, specifically in dermo-pharmacological products, due to their morphology, purity, high-water absorption, mechanical strength, and biocompatibility [13]. Among many bulk applications cellulase is strongly paving the way toward the biomedical sector. There is a high demand to develop new biomedical materials from natural polymers. Cellulase-based composites show potential in hydrogels, wound healing, drug delivery, bone tissue engineering, and even cancer treatment [13].

Immobilization of enzymes on a carrier can help with minimizing protein weaknesses, especially in enhancing their stability. Free, unbound enzymes usually operate under mild conditions, leading to quick function destabilization in industrial processes [14]. Most enzymes lose their activity during prolonged times of reaction or storage. The main reason lies in tertiary structure denaturation leading to active site poisoning. In addition, recovery of enzymes from the reaction mixture and their reuse can be very challenging [15]. The enzyme immobilization technique overcomes these limitations by improving specific properties. The choice of the most appropriate carrier material and the method of immobilization strongly depends on the type of catalytic activity of the biocatalysts as well as the condition of this activity [16].

Recently, there has been growing interest in the immobilization of enzymes on magnetic nanoparticles (MNPs) [17]. These nanoparticles possess unique properties, such as a high surface area, which facilitates efficient enzyme attachment. Additionally, their exceptional magnetic properties enable separation from complex mixtures using an external static magnetic field (e.g., magnets), thereby eliminating the need for complicated recovery processes. Immobilization can enhance catalytic reactions, often improving enzyme thermo- and pH stability [18]. Among the various magnetic nanomaterials, iron oxides are widely used for enzyme immobilization, including magnetite (Fe_3_O_4_, containing Fe^2+^ and Fe^3+^), hematite (α-Fe_2_O_3_), and maghemite (γ-Fe_2_O_3_). Iron oxides are particularly attractive for biological applications due to their relatively low production costs and toxicity, excellent magnetic properties, along with ease of functionalization with diverse organic moieties. These advantages make them highly suitable for immobilization processes [19]. However, unmodified magnetic nanoparticles have a strong tendency to form agglomerates. Additionally, magnetite, with its mixed-valence iron cations, is prone to oxidation when exposed to air or other oxidizing agents. To address these issues, it is crucial to modify their surface to prevent oxidation, improve stability, and keep sufficient magnetic properties [20,21]. Several strategies exist to anchor organic or make inorganic shells on the surface of MNPs [22]. The core-shell structures formed by different coatings ensure a robust bond with functional groups and allow further surface functionalization [23]. Moreover, the combination of the unique properties of magneto-plasmonic nanoparticles can lead to the development of new, advanced biomedical tools as shown by Mukha et al. [24].

The current study shows the cellulase from *Trichoderma reesei* immobilization process onto a Fe_3_O_4_@SiO_2_-NH_2_-Au magneto-plasmonic heterostructure. Cellulase was immobilized on the nanoparticles through covalent bonding using a 16-mercaptohexadecanoic acid (MHDA) linker characterized by the presence of a thiol (-SH) functional group with a high affinity toward metallic surfaces. The impact of various parameters on the enzymatic activity of the immobilized cellulase was evaluated, including pH and thermal stability. In addition, cellulase reusability was assessed by measuring its activity over multiple cycles.

## 2. Results and Discussion

### 2.1. Characterization of Heterostructures General Physicochemical Properties

**Figure 1:**
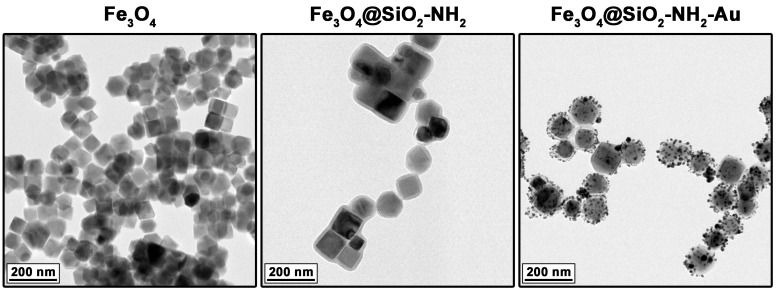
TEM imaging of the core magnetic particles (Fe_3_O_4_), hybrid intermediate compound (Fe_3_O_4_@SiO_2_-NH_2_), and final gold-containing magneto-plasmonic heterostructures (Fe_3_O_4_@SiO_2_-NH_2_-Au).

**Figure 2:**
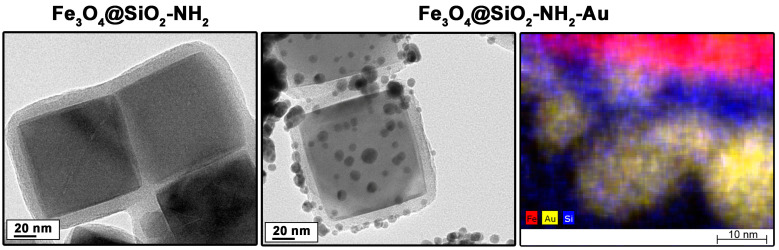
Magnification of the intermediate Fe_3_O_4_@SiO_2_-NH_2_ and final heterostructure Fe_3_O_4_@SiO_2_-NH_2_-Au TEM images with an element distribution map.

The size and morphology of the heterostructures were evaluated using the TEM technique, including the effects of each synthetic step toward them (see Figure 1). As observed, the core magnetic particles composed of Fe_3_O_4_ magnetite predominantly exhibited well-defined cubic shapes with an average size of 79 ± 12 nm. The mean thickness of the deposited amorphous silica shell containing NH_2_ functionalities (source APTES) was approximately 10 nm. The presence of amino-functional groups (with lone electron pairs) allowed specific chemisorption of gold cations that enabled their reduction and formation of the Au metallic nanoparticles (NPs) with an average size of 8 ± 3 nm (Figure 2). The Au NPs formed isolated islands with irregular morphology. The elemental map analysis confirmed the formation of Fe_3_O_4_@SiO_2_-NH_2_-Au heterostructures, with clearly distinguishable regions corresponding to the magnetite core particles, the silica shell, and the Au metallic particles located at the outer part of the silica interlayer (Figure 2, right side). These results indicate that the proposed synthetic approach is an effective tool for the fabrication of complex structures that integrate materials with diverse physicochemical properties (magneto-plasmonic feature). A more detailed analysis of the magneto-plasmonic heterostructures (with Au and Ag), regarding their structural features and other properties is available in our previous studies [25,26].

The primary objective of the heterostructure design was to enable fast, reliable, and cost-effective removal and reuse of cellulase-immobilized enzymes from the reaction medium facilitated by the magnetic core (Fe_3_O_4_). Meanwhile, the Au nanoparticles serve as active sites for the binding of high-affinity linkers (e.g., MHDA) containing thiol (-SH) functional groups [27] and o act as potential SERS probes for the possible identification of bioactive substances [28]. The surface-anchored MHDA was used as a linker, allowing bioconjugation of the chosen enzyme.

**Figure 3:**
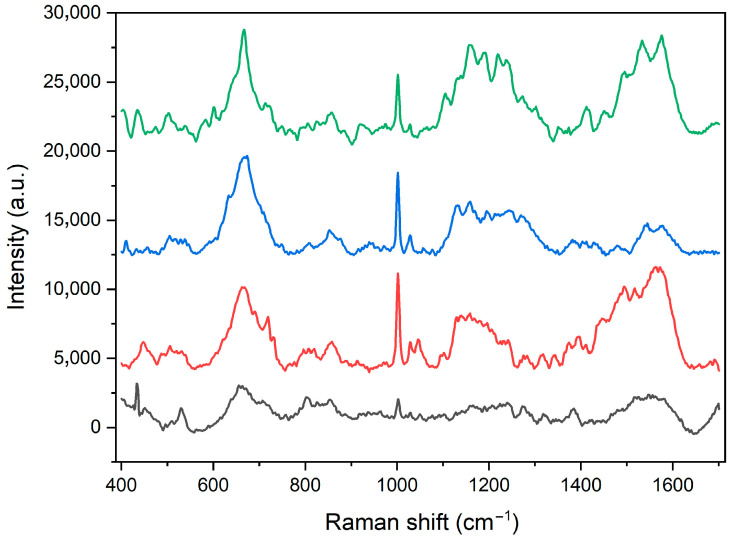
Raman spectra of Fe_3_O_4_@SiO_2_-NH_2_-Au-cellulase recorded at four different positions.

The effect of linker attachment and the bioconjugation of cellulase onto Fe_3_O_4_@SiO_2_-NH_2_-Au heterostructures was analyzed using Raman spectroscopy. The Raman spectra of heterostructures functionalized with MHDA molecules showed the presence of abundant spectral lines related to the following vibrations: Fe–O in magnetite form (~660 cm^−1^—A_1g_ and ~510 cm^−1^—T_2g_) [29], silica-derived bands (~610 cm^−1^ attributed to SiO_2_ vibrations, ~720 and ~760 cm^−1^ associated with Si–O–Si modes, and ~1560 cm^−1^ characteristic of Si–C) [30,31], N–H groups (~1240 cm^−1^, ~1380 cm^−1^, ~1570 cm^−1^), and C–N bonds (~1470 cm^−1^) [30]. The spectra of heterostructures with anchored MHDA linkers contained bands corresponding to the C–H vibrations of MHDA molecules (~1440 cm^−1^, ~1415 cm^−1^, ~1320 cm^−1^, ~1280 cm^−1^, ~1160 cm^−1^, ~810 cm^−1^). Bands associated with CO stretching vibrations (~1100 cm^−1^), C–C (~1060 cm^−1^), and C–S (~740 cm^−1^) were also observed [32,33]. The Raman spectra of Fe_3_O_4_@SiO_2_-NH_2_-Au-cellulase samples were recorded and presented in Figure 3. These spectra show vibration modes attributed to Fe–O, silica, and the MHDA linker. Additionally, in each recorded spectrum, bands originating from the protein molecules (cellulase enzyme) are clearly visible. The most characteristic peaks are related to phenylalanine (at 1002 cm^−1^) and tryptophan (at 1340 cm^−1^). Signals typical of other amino acids and amide bands were also detected (see Table 1). The results confirmed the effective attachment of the cellulase enzyme to the surface of the fabricated heterostructures through the MHDA linker. It should be noted that spectra recorded at different locations on the sample exhibited slight variations. This is attributed to the presence of gold nanoparticle islands on the heterostructure surface, which amplify signals through the generation of surface plasmons and the surface-enhanced Raman scattering (SERS) phenomenon. Although measurements were conducted under identical conditions, variations in the shape of the spectra may result from the localized amplification of certain signals due to the SERS effect.

### 2.2. Effects of Cellulase Heterostructure Immobilization

#### 2.2.1. Cellulase Binding Yield

**Figure 4:**
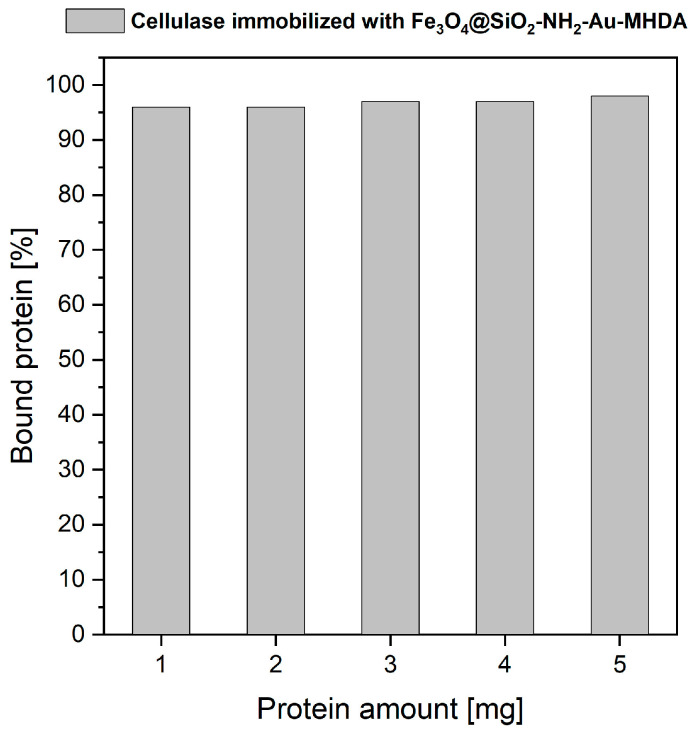
Binding yields of different amounts of cellulase to the Fe_3_O_4_@SiO_2_-NH_2_-Au heterostructure through the participation of the MHDA linker.

The cellulase from *Trichoderma reesei* was immobilized on the Fe_3_O_4_@SiO_2_-NH_2_-Au surface using the MHDA linker in accordance with the described procedure. The heterostructure capacity of enzyme binding was tested using the Bradford protein assay. As shown in Figure 4, the cellulase immobilization yield ranged from 96% to 98%, depending on the amount of enzyme used, suggesting near-complete conjugation of the enzyme with the MHDA linker for all tested protein quantities. We did not increase the cellulase concentration further since the 5:1 binding ratio is already high; however, it can be concluded that even more enzyme could potentially be loaded, as protein saturation was not observed during the 3.5-h incubation period.

**Figure 5:**
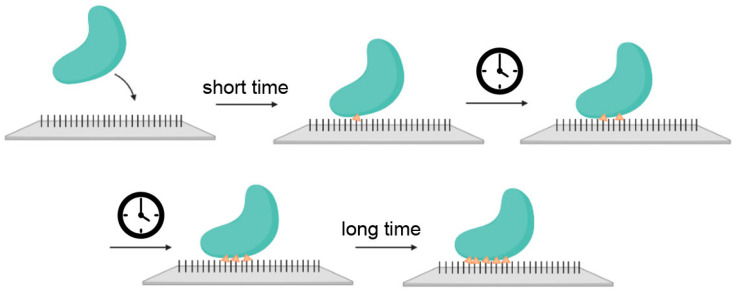
Time-dependent progressive multipoint covalent protein attachment to the carrier during enzyme immobilization.

Abraham et al. [37] conducted similar studies on the immobilization of cellulase derived from *Trichoderma reesei*, albeit with a completely different linker for protein conjugation, namely glutaraldehyde (GA). In their study, zinc-containing hematite nanoparticles with an average particle size of 40 nm were used as the carrier. Protein binding saturation was achieved at a 1:1 ratio after a 2-h incubation period at 25 °C. In our study, the incubation time was approximately 3.5 h. More importantly, we used a strongly bonded linker (MHDA) specific to gold particles with a size of 8 nm. This difference in protein-to-heterostructure immobilization ratios may be attributed to the smaller size and greater active surface area of the Au nanoparticles, as well as the high affinity of the MHDA linker for metal surfaces. Another important feature is that protein immobilization exhibits time dependence, leading to multipoint covalent attachment (see Figure 5). Consequently, the incubation time plays a significant role, and the use of longer chain linkers, such as MHDA, may result in a higher number of immobilized enzymes while also reducing protein rigidification [14,38]. These effects are responsible for and of great importance for enzyme stabilization.

#### 2.2.2. Effect of pH and Temperature on Cellulase Activity

**Figure 6:**
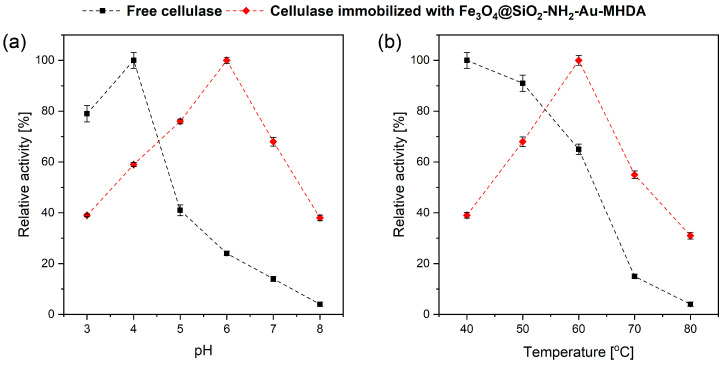
The pH (**a**) and temperature (**b**) effect on the activity of free and immobilized cellulase on Fe_3_O_4_@SiO_2_-NH_2_-MHDA heterostructure.

The ionization state of residues in the side chains of amino acids within an enzyme plays a pivotal role in maintaining its three-dimensional structure, which is essential for its functionality. Enzymes rely on their specific conformations to form active sites where substrates bind, and reactions are catalyzed. The ionization state of these residues affects the enzyme’s structural stability, the active site’s shape, and the charge distribution, all of which are critical for enzymatic activity. The pH is a primary environmental factor that directly influences the ionization state of these amino acid side chains. Each enzyme operates optimally within a specific pH range (optimal pH) where the ionization states of key residues are precisely tuned to facilitate efficient catalysis. Deviations from this optimal pH can lead to alterations in the degree of ionization of these residues, which in turn, can destabilize the enzyme’s conformation, inhibit substrate binding, or even lead to denaturation. Moreover, changes in pH can impact the formation of the enzyme–substrate complex, a critical intermediate in the catalytic process. For example, the ionizable groups in the active site or on the substrate may fail to interact correctly if the pH shifts, reducing the enzyme’s catalytic efficiency. In extreme cases, significant pH changes can permanently alter the enzyme structure through processes such as protonation or deprotonation, leading to a loss of function [39]. In Figure 6a, we present the effect of different pH values, ranging from 3.0 to 8.0, on the activity of free and immobilized cellulase on a heterostructure carrier. The optimal pH for free cellulase was 4.0, while the immobilized protein exhibited the highest relative activity at pH 6.0. A similar result regarding the optimal pH for immobilized cellulase was reported by Hegedus et al. [40]. However, in their study, activity was retained across a broad pH range. For free cellulase, incubation at pH values higher than 4.0 within the studied range resulted in a sudden decline in relative activity; at a reaction pH of 8.0, the enzyme retained only 4% of its initial activity. This suggests that deprotonation at the active sites of cellulase significantly impacts its catalytic activity [41]. The high activity of free cellulase at low pH values can be attributed to the fact that acidic conditions are the natural environment for cellulase-producing microorganisms, which supports and enhances its enzymatic activity [42]. On the other hand, immobilized cellulase with the Fe_3_O_4_@SiO_2_-NH_2_-Au-MHDA carrier exhibited the highest relative activity at pH 6.0 (Figure 6a). Thus, the pH optimum for the immobilized cellulase was shifted by two pH units compared to the free enzyme. However, at pH values above and below the optimum, the immobilized cellulase displayed reduced activity. Notably, the immobilized cellulase demonstrated greater tolerance to higher pH values compared to the free enzyme.

At a pH value of 7.0, the relative activity of immobilized cellulase was maintained at 64% of its initial activity, compared to only 14% for free cellulase. When the pH was increased to 8.0, the relative enzymatic activity of immobilized cellulase decreased to 38%, while the free enzyme retained merely 4% of its initial activity. This difference can be attributed to the stabilization of the cellulase’s molecular conformation upon binding to the carrier, which may limit or eliminate the direct effects of unfavorable factors, such as pH changes. Such stabilization protects the enzyme and reduces its rate of inactivation in varied environments. Most studies indicate that the optimal pH for the activity of immobilized *Trichoderma reesei* cellulase is 5.0 [43,44]. Thus, the observed improvement in the stability of immobilized cellulase at higher pH values is significant. A similar pH tolerance profile was reported by J.S. Lima et al. for *Trichoderma reesei* cellulase immobilized on Fe_3_O_4_ alone nanoparticles [45].

Temperature significantly impacts enzyme function by affecting molecular kinetic energy and enzymatic reaction rates. Enzymes have an optimal temperature for maximum activity, beyond which activity declines due to structural changes and catalytic inefficiency. Sensitivity varies with enzyme origin and environmental adaptation, with psychrophilic enzymes favoring the cold, while thermophilic enzymes prefer elevated temperatures. Their activity is shaped by the thermal equilibrium between active and inactive states. Therefore, substrate availability and diffusion rates further complicate the temperature–kinetic relationship [46]. Exposure to temperatures higher than optimal causes its gradual denaturation and loss of catalytic abilities. The enzymatic activity of free and immobilized cellulase on Fe_3_O_4_@SiO_2_-NH_2_-Au-MHDA heterostructures was tested at different temperatures of 40–80 °C (Figure 6b).

The results indicate that the activity of free cellulase follows a different trend compared to immobilized cellulase. Free cellulase exhibits maximum activity at 40 °C, but its relative activity decreases sharply beyond this temperature, reaching only 4% at 80 °C. This behavior highlights the sensitivity and rapid denaturation of free cellulase, particularly above 60 °C. The temperature increase may break numerous bonds that stabilize the active center and structure of the enzyme. In contrast, immobilized cellulase demonstrates a distinct thermal stability profile. It achieves maximum enzymatic activity at 60 °C, indicating an optimal operating temperature 20 °C higher than that of free cellulase. This enhanced thermal stability is attributed to the MHDA linker, which forms stable amide bonds with the conjugated protein. Such covalent bonding reduces the conformational flexibility of the protein, protecting it from heat-induced distortion or damage, resulting in enhanced heat resistance compared to the free enzyme. This kind of structure stabilization may also require a higher activation energy of the molecule to reorganize for binding with the substrate, as well as higher energy to disorganize the enzyme structure. Numerous studies on the immobilization of *Trichoderma reesei* cellulase using nanoparticles reported optimal activity at 50 °C [44,45,47]. However, the present study demonstrates that the thermal stability of cellulase is improved by an additional 10 °C, confirming the effectiveness of the proposed immobilization in enhancing the enzyme’s thermo resistance.

#### 2.2.3. Cellulase Thermal Stability over Time

**Figure 7:**
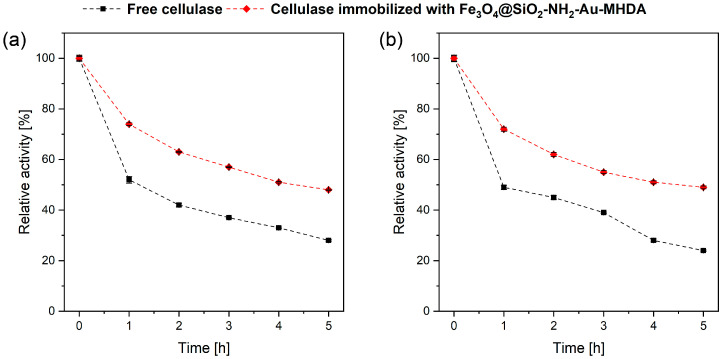
Cellulase thermal stability as a function of time at the optimal temperature for the free enzyme at 40 °C (**a**) and immobilized cellulase at 60 °C (**b**) with direct comparison of the activity of both enzyme forms for each temperature.

The operational stability of immobilized enzymes is crucial for cost reduction during practical applications. The stability of free and immobilized cellulase was evaluated by measuring their activity at 1-h intervals over 5 h, under optimal temperature conditions for the free enzyme (40 °C, Figure 7a) and the immobilized enzyme (60 °C, Figure 7b), as determined by previous experiments. For free cellulase, a sharp decrease in activity was observed after the first hour at both 40 °C and 60 °C, with further activity dropping to 52% and 49% of its initial value, respectively. Over the subsequent hours, activity gradually decreased, reaching 28% and 24% of the initial value after 5 h of incubation. In contrast, the immobilized cellulase exhibited similar trends in relative activity but maintained higher activity levels than the free enzyme. After 5 h of incubation, cellulase immobilized on Fe_3_O_4_@SiO_2_-NH_2_-Au preserved 48% of its initial activity at 40 °C and 49% at 60 °C. These results indicate that the immobilized enzyme demonstrates relatively stable activity over time. The slower loss compared to the free enzyme highlights the superior stability of the immobilized cellulase.

**Figure 8:**
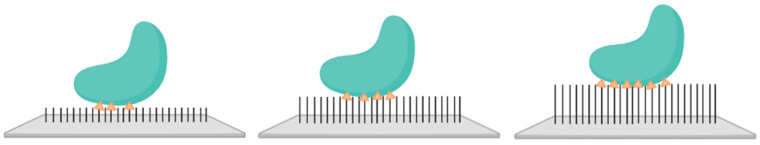
Effect of the linker chain length on enzyme stabilization through multipoint covalent attachment.

We conclude that the conjugation of cellulase with MHDA contributes significantly to enzyme stabilization. The carboxylic acid with thiol functionality, characterized by its relatively long carbon chain, offers potential benefits. Longer linkers can provide additional flexibility, facilitating the formation of multiple covalent bonds with the protein (see Figure 8). This multipoint attachment likely enhances the stabilization of the enzyme and improves its resistance to adverse conditions [14,38].

On the other hand, the length and flexibility of the linker can positively influence substrate accessibility to the immobilized protein. Consequently, even with a high protein loading on the carrier, the enzyme can adopt a favorable conformation, ensuring better interaction with the substrate while maintaining stability. Overall, the results indicate that cellulase stability over time significantly improved after immobilization on Fe_3_O_4_@SiO_2_-NH_2_-Au. The stability of cellulase immobilized on nanoparticles has been extensively analyzed by various research groups, confirming its high stability and potential for effective protein storage [37,41,48].

### 2.3. Reusability of the Cellulase Immobilized Enzyme on Heterostructures

**Figure 9:**
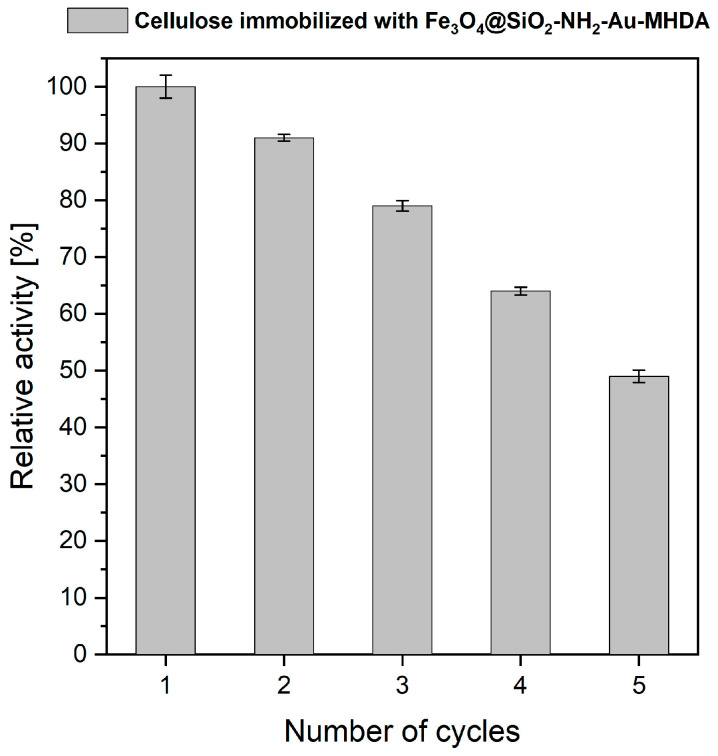
Reusability of the cellulase immobilized on Fe_3_O_4_@SiO_2_-NH_2_-Au-MHDA carrier.

The operational stability of the immobilized enzyme, combined with the use of a magnetic carrier, offers the potential for effective cellulase recycling, thereby reducing operating costs. One of the most valuable properties of immobilized systems is the preservation of catalytic activity after immobilization, which is crucial for practical applications. In this study, the cyclic use of immobilized cellulase was assessed by measuring its activity over successive hydrolysis cycles (Figure 9).

The results demonstrated a gradual decrease in cellulase activity with each cycle on a carrier, with an approximately 10–15% activity loss per cycle. The Fe_3_O_4_@SiO_2_-NH_2_-Au cellulase immobilized heterostructure retained 49% of its original activity after five reuse cycles. Several factors may contribute to the partial loss of immobilized enzyme activity, including enzyme desorption from the carrier, inevitable enzyme deactivation, structural modifications, and partial loss of the enzyme carrier during recovery [38]. In this study, we observed increased adhesion of the composite to the plastic surfaces of laboratory equipment. Protein adhesion can occur when proteins contact hydrophobic surfaces, such as plastics. Additionally, uneven distribution of the coating on the magnetic core may lead to increased self-aggregation, causing conformational changes and altered interactions between immobilized cellulase molecules. These effects could reduce enzyme activity during subsequent cycles and result in less efficient composite recovery. In other studies, immobilization of *Trichoderma reesei* cellulase on styrene-maleic anhydride (SMA) copolymer nanoparticles retained 85% of enzyme activity after 10 hydrolysis cycles [49]. J.S. Lima et al. reported that the catalytic efficiency of cellulase immobilized on similar Fe_3_O_4_ nanoparticles dropped to approximately 31% of its initial value after eight reaction cycles [45]. In studies involving modified magnetic nanoparticles, such as Fe_3_O_4_-chitosan, high cellulase activity was maintained even after several hydrolysis cycles [48,50]. Our results suggest that immobilized cellulase on magneto-plasmonic heterostructures, such as Fe_3_O_4_@SiO_2_-NH_2_-Au, can be effectively reused for several reaction cycles. The primary advantage of this system lies in the simple and rapid recovery of cellulase after each hydrolysis cycle through efficient magnetic separation. Further research will be necessary, particularly focusing on the optimization of linkers and carriers, to improve stability and reusability.

## 3. Materials and Methods

### 3.1. Synthesis of Magneto-Plasmonic Heterostructures

**Figure 10:**
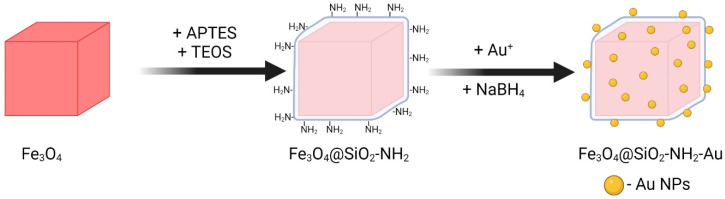
Scheme of the multistep fabrication process of Fe_3_O_4_@SiO_2_-NH_2_-Au heterostructures combining magnetic and gold nanoparticles.

The synthetic protocol for the fabrication of magnetite heterostructures decorated with gold nanoparticles (Fe_3_O_4_@SiO_2_-NH_2_-Au) was divided into the following steps (Figure 10): (I) the preparation of seed magnetic nanocubes via the thermal decomposition of an iron precursor in a high-boiling-point organic solvent, (II) the deposition of an amorphous silica shell containing amino groups onto the surface of the seed Fe_3_O_4_ nanoparticles using a wet-chemistry technique, and (III) the chemisorption of gold species followed by the reduction of gold ions [25].

#### 3.1.1. Synthesis of Cubic Magnetite Particles

Cubic magnetite particles were prepared by combining 2 mmol of Fe(acac)_3_ (99.7%, Thermo Fisher Scientific, Gdańsk, Poland), 4 mmol of oleic acid (OA, 90%, Sigma-Aldrich, Poznań, Poland), and 10 mL of dibenzyl ether (BE, 98%, Sigma-Aldrich, Poznań, Poland). All chemical handling was performed in an acrylic glovebox (GS Glove Box Systemtechnik GMBH P10R250T2, Malsch, Germany) under a nitrogen (N_2_) atmosphere. The iron precursor was dissolved in BE in the presence of OA. The solution was transferred to a three-neck flask equipped with a mechanical stirrer, a temperature controller with a Pt-100 sensor (LTR 2500, Juchheim, Bernkastel-Kues, Germany), a heating mantle, a reflux column, and inert gas inlet and outlet ports. The reaction mixture was degassed at room temperature for 1 h and subsequently heated to 285 °C, where it was maintained for 30 min. Afterward, the reaction was cooled to room temperature. The resulting black product was separated from the mother liquor and purified through multiple washing and centrifugation steps using a hexane/acetone/ethanol mixture (all reagents pure for analysis, Chempur, Piekary Śląskie, Poland). The purified particles were suspended in fresh ethanol and stored in a laboratory refrigerator for future use.

#### 3.1.2. Silica Shell Deposition with NH_2_ Functionalities

A silica shell containing NH_2_ functional groups was deposited as follows: 50 mg of Fe_3_O_4_ cubic particles were dispersed in hexane and sonicated for 2 h at room temperature. Subsequently, 45 mL of additional hexane (pure for analysis, Chempur, Piekary Śląskie, Poland) and 2 mL of IGEPAL CO-520 (Sigma-Aldrich, Poznań, Poland) were added to the dispersion and sonicated for 15 min to enhance particle dispersion. Afterward, 0.4 mL of ammonia (25% solution, 99%, Honeywell, Warszawa, Poland) was added to the mixture, which was then sonicated for 15 min. The resulting mixture was transferred to a glass flask attached to a mechanical stirrer, and 100 µL of TEOS (tetraethoxysilane, 99.9%, Thermo Scientific, Warszawa, Poland) was added under vigorous stirring for 1 h. Another 100 µL each of TEOS and APTES ((3-aminopropyl)triethoxysilane, 99%, Sigma-Aldrich, Poznań, Poland) were added, and the reaction mixture was stirred continuously for 24 h. The resulting Fe_3_O_4_@SiO_2_-NH_2_ hybrid was separated magnetically, washed several times with an acetone/ethanol mixture, and resuspended in ethanol.

#### 3.1.3. Decoration with Gold Nanoparticles

For the final step, 2 mg of the prepared Fe_3_O_4_@SiO_2_-NH_2_ particles were dispersed in 20 mL of H_2_O under sonication for 10 min. The particle dispersion was placed in an ice bath (8–10 °C), and 314 µL of 0.012 M HAuCl_4_ (99.99%, Sigma-Aldrich, Poznań, Poland) was added dropwise under constant stirring. The temperature was then raised to 23–25 °C, and 200 µL of 0.012 M NaBH_4_ (a strong reducing agent, 99%, Thermo Scientific, Warszawa, Poland) was added. After 15 min, the purification process of Fe_3_O_4_@SiO_2_-NH_2_-Au heterostructures was started, involving several washing and magnetic separation cycles using water and ethanol as dispersing media.

#### 3.1.4. Anchor of the MHDA to Magneto-Plasmonic Heterostructure and Conjugation with the Cellulase Enzyme

**Figure 11:**
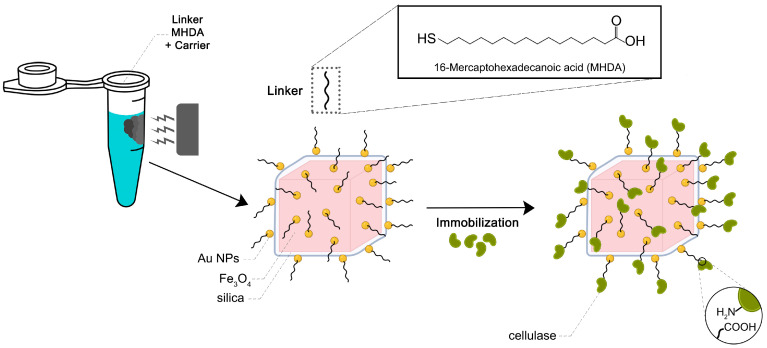
Scheme of the cellulase immobilization through the MHDA linker on the Fe_3_O_4_@SiO_2_-NH_2_@Au heterostructures.

The cellulase immobilization process (Figure 11) was carried out following the protocol by Wojnarowska et al. [33], with some modifications. Specifically, 1 mg of Fe_3_O_4_@SiO_2_-NH_2_-Au nanoparticles suspended in ethanol was incubated for 24 h at 4 °C with 50 µL of 5 mM 16-mercaptohexadecanoic acid (MHDA, 98%, Sigma-Aldrich, Poznań, Poland). The resulting product was sequentially washed first with 500 µL of N,N-dimethylformamide (DMF, 99%, Sigma-Aldrich, Poznań, Poland) and then with 500 µL of an activation solution containing 20 mM N-cyclohexyl-N′-(2-morpholinoethyl)carbodiimide methyl-p-toluenesulfonate (CMC, 95%, Sigma-Aldrich, Poznań, Poland), 20 mM pentafluorophenyl-4-vinyl benzoate (PFP, 99%, Sigma-Aldrich, Poznań, Poland), and 20 mM N,N-diisopropylethylamine (DIPEA, 99%, Sigma-Aldrich, Poznań, Poland) in DMF. Between each washing step, the nanoparticles were sonicated and magnetically separated. After washing, the carrier was resuspended in 100 µL of the activation solution, sonicated to ensure uniform dispersion, and incubated for 30 min at 25 °C with shaking at 850 rpm. Following activation, the carrier was washed twice with 500 µL of DMF and resuspended in 100 µL of DMF. Next, an enzyme solution containing 1 mg of protein was added to the activated nanoparticle suspension and incubated for 3 h at 25 °C with shaking at 850 rpm. After this period, the supernatant was collected for further analysis, and the resulting cellulase–MNP complex was washed three times with 300 µL of citrate buffer (pH 6.0) to remove the unbound enzyme. Each washing step involved sonication and magnetic separation. Finally, the biofunctionalized nanoparticles were resuspended in 1 mL of citrate buffer (pH 6.0) and stored for subsequent analysis.

### 3.2. Characterization of Physicochemical Properties of Nanomaterials

The particle size and morphology of the Fe_3_O_4_ heterostructures were evaluated using transmission electron microscopy (TEM) with a Tecnai Osiris X-FEG (FEI Company, Hillsboro, OR, USA) operating at 200 kV. Elemental distribution maps were obtained using high-angle annular, dark-field scanning TEM (HAADF-STEM) and energy-dispersive spectroscopy (EDS). For TEM imaging, sample preparation involved depositing a droplet of the heterostructures’ ethanol colloidal suspension onto a carbon-coated 200-mesh Cu grid (EM Resolutions, Newcastle, UK), followed by overnight drying under dust-free conditions. For details on the structural properties of the heterostructures, including X-ray powder diffraction and FTIR spectroscopy, readers are kindly referred to our previous publication [25].

Raman spectra were recorded using an InVia Micro Raman spectrometer (Renishaw, Wotton-under-Edge, Gloucestershire, UK) coupled with a Leica DM 2500M microscope and a 785 nm laser line as the excitation source. The laser output power was set at 0.5 mW to avoid thermal effects and prevent chemical or structural alterations to the sample. The analyzed samples were placed on quartz discs and allowed to dry slowly. Baseline correction was performed during data processing.

### 3.3. Characterization of the Cellulase Heterostructure Immobilization Conditions

#### 3.3.1. Cellulase Binding Yield, Effect of pH and Temperature

To evaluate the influence of cellulase concentration on immobilization effectiveness, different amounts of enzyme were used for the bioconjugation process via the surface MHDA linker. Specifically, 1, 2, 3, 4, and 5 mg of cellulase were tested, and the immobilization process was performed by using the same operations as written in the section devoted to the cellulase conjugation to MHDA. Protein concentrations were measured using the Bradford assay method [51]. The 96-well plate was loaded with 250 µL of the reagent and 10 µL of the standard solution/test sample per well. The mixture was incubated for 5 min, and then the samples were read at 595 nm. The enzyme immobilization yield was determined using the following formula:

(1) $$binding\;yield=(\frac{M_{0}-M_{1}}{M_{0}})100\%,$$

where *M*_0_ represents the total amount of protein added to the mixture during the conjugation process, and *M*_1_ is the amount of unbound protein remaining in the supernatant.

To investigate the pH effect on the activity of immobilized cellulase resulting enzyme-heterostructure constructs were incubated in McIlvaine’s buffer (pH 3.0; 4.0; 5.0; 6.0; 7.0; 8.0) by performing the enzymatic activity test at 50 °C for 15 min. The temperature dependence on immobilized cellulase activity was assessed after incubation at various temperatures: 40, 50, 60, 70, and 80 °C. The obtained results were compared to those for free enzyme and tested under the same conditions in a citric buffer (pH 6.0). All assays were conducted in triplicate.

#### 3.3.2. Cellulase Thermal Stability

To assess the stability of free and immobilized cellulase over time, the activity of the cellulase heterostructures was measured hourly for 5 h under optimal temperature conditions, as determined during the evaluation of the activity’s temperature dependence.

### 3.4. Assessment of the Catalytic Activity of Immobilized Cellulase

Enzyme activity was assessed by measuring the release of p-nitrophenol (4-NP), produced from the hydrolysis of the substrate 4-nitrophenyl-β-D-glucopyranoside (pNPG), which contains a chromogen. The release of the 4-NP should follow the rule that one unit of enzyme activity is equivalent to the amount of enzyme required to produce 1 µmol p-nitrophenol/min. For the activity assay, 2 mM solution of pNPG in citrate buffer (pH 6.0) was prepared. The reaction mixture consisted of 900 µL of the 2 mM pNPG solution and 100 µL of the test sample containing either free or immobilized enzyme. For the blank control, 100 µL of citrate buffer (pH 6.0) was used instead of the enzyme sample. For pH optimization experiments, buffers of varying pH were substituted for the citrate buffer (pH 6.0). All solutions were vortexed and incubated for 30 min at 40 °C. To stop the reaction, 2 mL of 1 M Na_2_CO_3_ (99.5%, Sigma Aldrich, Poznań, Poland) was added, and absorbance was measured at 450 nm. The concentration of released p-nitrophenol was determined using a standard curve prepared from 4-NP solutions of known concentrations.

### 3.5. Protocol for Enzyme Reusability on Heterostructures

To evaluate the reusability and effectiveness of the immobilized cellulase, its activity was measured every hour over a 5-h period, using the same cellulase heterostructure in each cycle. The assays were performed at 60 °C and pH 6.0. After each cycle, the cellulase-MNP composite was recovered using a magnetic separation, rinsed, resuspended in 100 µL of buffer, and combined with 900 µL of fresh substrate solution. The mixture was then incubated under the same conditions for the subsequent cycle.

## 4. Conclusions

Fe_3_O_4_@SiO_2_-NH_2_-Au heterostructures were successfully synthesized with well-defined cubic Fe_3_O_4_ cores (79 nm) and a 10 nm silica shell. Gold nanoparticles (8 nm) were fabricated through chemisorbed gold cation reduction on the silica surface. For cellulase conjugation to the heterostructure surface, the MHDA linker was employed. The relatively long carbon chain of the linker provided enzyme flexibility, while its thiol functional groups ensured strong binding with the gold nanoparticles and overall complex structure. Using Raman spectroscopy, we demonstrated that gold-containing heterostructures can effectively serve as SERS probes for signal amplification. Furthermore, we provided evidence for the functionalization of the heterostructures with the MHDA linker and subsequent bioconjugation with an enzyme, confirmed by the presence of characteristic vibrational modes in the recorded Raman spectra. We have also shown that the chosen synthetic approach is effective for fabricating a complex system with diverse physicochemical properties (magneto-plasmonic-catalytic), enabling efficient cellulase immobilization with potential applications in enzyme recovery and sensing. The use of magnetic carriers facilitates easy and rapid recovery of immobilized enzymes through magnetic separation, which could contribute to the minimization of operational costs. The immobilization of cellulase on Fe_3_O_4_@SiO_2_-NH_2_-Au-MHDA shifted its optimal pH from 4.0 (free enzyme) to 6.0 and improved its tolerance to higher pH values, retaining 64% and 38% activity at pH 7.0 and 8.0, respectively. This enhanced stability was attributed to structural stabilization provided by the carrier. Additionally, the immobilized cellulase exhibited improved thermal stability, with an optimal temperature of 60–20 °C higher than the free enzyme. At 80 °C, the immobilized cellulase kept significantly more activity compared to the nearly inactive free enzyme. Future work should focus on further optimizing such linkers and multifunctional carriers to improve the stability, efficiency, and reusability of immobilized cellulase systems. Optimization of linkers should focus on varying their carbon chain length to examine the impact on protein flexibility or rigidity within the same thiol-containing carboxylic organic ligands, which can significantly influence enzyme activity. The potential use of more complex linkers with multiple carboxylic functions, along with high affinity for metal surface thiol groups, should be explored to enable more effective multivalent covalent attachment of proteins. Additionally, core particle size reduction should be considered to study the effect of increased linker anchoring, driven by the enhanced surface area and the potential for higher protein conjugation. Optimization of the number of Au nanoparticles could also be investigated, as this would increase the density of thiol-carboxylic ligand anchoring sites. Our findings are consistent with previous studies, confirming the high stability and storage potential of cellulase immobilized on nanoparticles.

## Figures and Tables

**Table 1:** The Raman peak positions and band identification of cellulase enzyme vibration modes [33,34,35,36].

Peak Positions (cm^−1^)	Band Identification
1560	tryptophan: ν(C=C)
1530	amide II: ν(C-N), δ(N-H)
1446	σ(CH2), σ(CH3)
1342	tryptophan: CH2/CH3 wagging, twisting and/or bending mode
1270	amide III (ν(C-N), δ(N-H), ν(CH3-C)), ν(P=O)(FAD)
1130	δ(CCH), ν(CC), ν(C-N)
1057	ν(C-N), ν(C-O)
1029	phenylalanine: δ(C-H)
1002	phenylalanine: ring breathing
914	proline/valine/protein backbone (α-helix conformation): ν(C-C)
871	hydroxyproline, tryptophan: β(CH) ring
857	tyrosine: ν(CC) ring
820	tyrosine: ν(CC) ring, β(CH) ring, γr(CH2)
765	tyrosine: ring breathing, γ(N-H),
644	amid IV (δ(O=C-N)), δ(CCN)

## Data Availability

The data are available from the corresponding authors on reasonable request (R.P. physicochemical characterization (TEM, EDS, and Raman spectra; D.B. all data regarding linker attachment, cellulase immobilization and parts regarding enzyme activity).
